# Treatment burden among patients with heart failure attending cardiac clinic of Tikur Anbessa Specialized Hospital: an explanatory sequential mixed methods study

**DOI:** 10.1038/s41598-022-23700-0

**Published:** 2022-11-07

**Authors:** Minimize Hassen, Desalew Mekonnen, Oumer Sada Muhammed

**Affiliations:** 1 Department of Clinical Pharmacy, School of Pharmacy, College of Medicine and Health Sciences, Wollo University, Dessie, Ethiopia; 2 Department of Internal Medicine, School of Medicine, College of Health Sciences, Addis Ababa University, Addis Ababa, Ethiopia; 3 Department of Pharmacology and Clinical Pharmacy, School of Pharmacy, College of Health Sciences, Addis Ababa University, Addis Ababa, Ethiopia

**Keywords:** Cardiology, Health care, Medical research

## Abstract

Emerging evidences hypothesized that patients with heart failure are susceptible to experience treatment burden. Despite this fact, no attempt was made so far to address this neoteric construct in the sub-Saharan African health care context. Hence, this study aimed to assess patients’ and health care providers’ perspectives on how to decrease treatment burden among patients with heart failure attending the adult cardiac clinic of Tikur Anbessa Specialized Hospital (TASH). An explanatory sequential mixed methods study was conducted at the adult cardiac clinic of TASH, Addis Ababa, Ethiopia from August 01 to September 30, 2021. Simple random and purposive sampling techniques were employed to select participants for quantitative and qualitative studies, respectively. Descriptive analysis was done to summarize the quantitative data. Logistic regression analysis was performed to identify predictors of treatment burden. *P* value < 0.05 was considered to declare statistical significance. Qualitative data were analyzed by using thematic analysis. A total of 325 patients were enrolled in the quantitative study. For the qualitative study, 14 patients and 11 health care providers (five nurses and six medical doctors) were included. Participants mean global Treatment Burden Questionnaire (TBQ-15) score was 27.22 ± 19.35. Approximately 12% (n = 38) patients indicated high treatment burden (TBQ-15 global score ≥ 59) with a median global score of 63(60–69). Higher education level (adjusted odds ratio [AOR] = 6.66, 95% confidence interval [CI]: 1.16–38.43), presence of two and more comorbidities (AOR = 2.74, 95%CI: 1.02–7.39), daily intake of more than five pills (AOR = 7.38, 95%CI: 2.23–24.41), poor medication availability (AOR = 3.33, 95%CI: 1.33–8.36), presence of medication adverse effects (AOR = 4.04, 95%CI: 1.63–10.03), and higher monthly cost of medication (AOR = 5.29, 95%CI: 1.46–19.18) were predictors of treatment burden. Patients and healthcare providers' propositions were primarily focused on improving self-care management, structural organization of the clinic and hospital, and healthcare system provision. Our findings demonstrated that a substantial proportion of patients faced low levels of treatment burden. This study unveiled that improving self-care management, structural organization of the clinic, and healthcare system provision had paramount importance to reducing treatment burden. Hence, factors affecting treatment burden should be considered when designing tailored healthcare interventions for patients with heart failure.

## Introduction

Chronic heart failure (CHF) is a chronic progressive cardiovascular disease with multifaceted etiologies, affecting nearly 2% of the adult population living in westerns, ordinarily increasing to 5–9% in the age above 65 years^1^. Unfortunately, CHF is a complex health problem that requires ample self-efficacy and strict adherence to the lifelong complex treatment protocol, uninterrupted engagement in health care, multiple lifestyle changes, regular symptom monitoring, and adaptive coping skills till the final days of life^2–4^.

Furthermore, managing CHF on its own is a perpetual and demanding job of work that is frequently complicated by comorbidities that produce treatment onerousness^5^. As a result, patients with HF often face prodigious barriers in their care and are highly susceptible to experience the burden of treatment. Regrettably, these burdens remain inconspicuous to health care providers^6,7^.

Treatment burden refers to the workload that a patient must manage to take care of their health and its impact on the patient’s daily life^4^. It is an indispensable clinical neoteric concept that needs to be addressed in patients with HF. This is because, it contributes to the occurrence of lower rates of adherence to prescribed treatments and self-care which will lead to worse clinical outcomes such as more hospitalizations, higher mortality, poorer quality of life, and symptom recurrence^6,8^.

Although the burden associated with a chronic illness is well articulated, the extent to which they are associated with the treatment and self-management of the disease remains poorly defined. As a result, available studies done^4,9–13^ so far to assess treatment burden in chronic illnesses are quite limited. Besides, most of them are qualitative and primarily focused on specific medical conditions such as diabetes mellitus, cancer, and HIV/AIDS. Despite its uttermost importance in the self-management of heart failure, little is investigated about this phenomenon.

Evidences^13–15^ indicated that treatment burden has many generic features, however, it is also highly likely to differ between specific countries and specific diseases. This is mainly because it is a contextual construct that depends on varying factors^13,16^. Hence, findings from previous qualitative studies conducted in different developed countries cannot be directly extrapolated into sub-Saharan Africa, including Ethiopian, healthcare context and thus needs further exploration.

Until today, only a few studies^13,17^ have explored patients’ propositions to improve their care and reduce treatment burden. However, these studies are conducted in developed countries and thus could not be generalized to the Ethiopian health context. Moreover, the health care provider's perspective on reducing treatment burden is a neglected area of concern that is often missed in the majority of the studies. So, the current study was designed to fill these gaps of knowledge. Thus, this study aimed to assess patients’ and health care providers’ perspectives on how to decrease treatment burden among ambulatory patients with CHF attending the adult cardiac clinic of TASH.

## Methodology

### Study design, study setting, and study period

This study employed an explanatory sequential mixed methods study, wherein a quantitative study (cross-sectional) was followed by a qualitative study. The study was conducted at the ambulatory cardiac clinic of TASH, Addis Ababa, Ethiopia from August 01 to September 30, 2021. TASH is the largest tertiary care, specialized, referral, and teaching hospital in the country that is owned by the government and established in 1973. TASH has 51 specialty out-patient clinics, serving 500,000 patients annually^18^. The cardiac clinic is part of the specialty clinics that offers comprehensive cardiac care including treatment and follow-up. It provides service four days per week (Monday, Tuesday, Wednesday, and Friday). On average, 70 HF patients came for follow-up each day.

### Study participants

All adult ambulatory patients with HF that fulfilled the inclusion criteria within the study period were included in the study. Patients were eligible for enrollment in this study if they were at least 18 years of age, diagnosed with CHF as confirmed by echocardiography with or without other comorbidities for at least 6 months before the study, and have had regular follow-up at the cardiac clinic of TASH, under HF treatment for at least 6 months and who can complete a written consent form. Critically ill patients who could not stand the interview and patients with cognitive impairment that could interfere with understanding the questionaries were excluded. Health care providers were selected for qualitative interview based on their work experience (minimum of 3 years) and familiarity in heart failure management, and willingness to participate in the study.

### Sample size determination and sampling technique

The sample size was calculated based on a single population proportion formula using 95% confidence level, 5% margin of error, 50% proportion of treatment burden, expected number of source population in the study period (N = 2240), and 5% non-response rate. Accordingly, a final sample size of 344 patients with CHF was calculated. Of which, 19 patients were excluded and a total of 325 patients were included in the final analysis.

A simple random sampling technique was employed to select study participants who fulfilled the stated inclusion criteria for the quantitative study. The nursing appointment logbook was used as a sampling framework. Patients were recruited randomly into the study during their appointment for medication refilling. For the qualitative study, the purposive sampling technique was employed to gather in-depth information from the study participants and health care providers. Accordingly, 14 patients (7 female) and 11 health care providers (4 male and 7 female) were selected for the qualitative study. The health care providers were comprised of five experienced nurses and six medical doctors (1 senior specialist, 3 fellowship residents, and 2 chief R3 residents). Patients were selected for in-depth interviews based on their TBQ global score whereas health care providers were selected for key informant interviews based on their work experience and familiarity in the management of heart failure. Eligible patients were invited by an oral invitation from the nurse at the outpatient clinic by briefly explaining the study. Health care providers were invited by an invitation letter explaining the study. Participants who accepted the invitation were contacted by telephone to arrange a convenient date and time for the interview.

### Study variables

#### Dependent variable

Treatment Burden.

#### Independent variables

(1) Sociodemographic characteristics include age, sex, place of residence, occupation status, monthly income level, living conditions, marital status, educational level, and smoking habit (2) Clinical characteristics include duration of CHF, stage of CHF, presence and number of comorbidities, travel time, number of appointments, number of hospitalizations during the past 1 year, knowledge about CHF, and health literacy (3) Treatment-related characteristics include total number of prescribed medications/pills, source of medication, medication type, cost of medication, availability of medications, and adverse effects.

### Data collection instrument and procedure

The study was conducted in two phases. In phase I, quantitative data on the patient's characteristics and level of treatment burden was collected via well-validated questionnaires. Treatment Burden Questionnaire (TBQ-15) was employed to determine the level of treatment burden of patients with HF. Before the administration of TBQ, each participant completed a questionnaire developed to gather information on the sociodemographic, disease, and treatment-related characteristics. Participants’ medical records were also reviewed to supplement further clinical data such as presence, type and number of comorbidities, hospitalization history, baseline ejection fraction (EF), and New York Heart Association (NYHA) functional class of heart failure. Two nurses administered the 15-item TBQ for each eligible participant. The quantitative data collection process lasted for 15–20 min on average.

TBQ-15 is a well-validated universal psychometric instrument designed to measure treatment burden associated with multiple chronic conditions. It is composed of 15 items that assess domains or arenas such as troubles associated with taking medicine, self-monitoring, adapting to a certain lifestyle, keeping up with laboratory tests, doctors’ appointments, social life, and organization and administrative burden. TBQ-15 is comprised of five key dimensions: medication-related burden, administrative-related burden, financial-related burden, lifestyle change-related burden, and social life-related burden^19^. The instrument responses are rated using a Likert-type scale ranging from 0 (not a problem) to 10 (a big problem). The TBQ global score is generated through the summation of each item score to a maximum score of 150 points.

**Figure 1:**
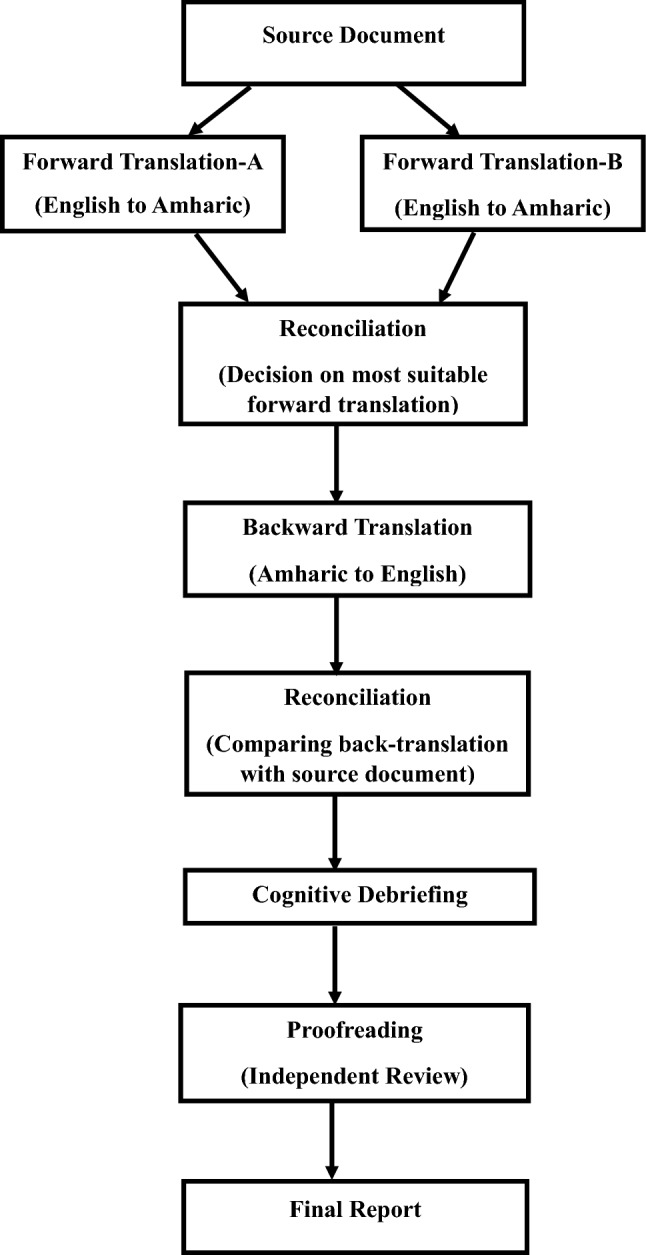
Summary of the steps followed for the translation and cultural adaption of the TBQ into Amharic for Ethiopia context (based on Linguistic Guidance for COA developed by MRT and ISPOR).

Permission to use and authorization to translate the TBQ was obtained from the original developer through Mapi Research Trust (MRT). A rigorous translation approach was applied as accorded by the linguistic validation guidance of a Clinical Outcome Assessment (COA) for translation and cultural adaption of patient-reported outcome measures developed by the MRT and International Society for Pharmacoeconomics and Outcomes Research (ISPOR) (Fig. 1). A detailed report of the translation process along with the final validated Amharic for Ethiopia version of TBQ-15 was successfully uploaded to the MRT database and can be accessed via http://www.mapi-trust.org. The Cronbach alpha for the Amharic for Ethiopia version of TBQ-15 was 0.761. However, except for internal consistency, no further psychometric testing was done.

In phase II, qualitative data on patients and health care providers' perspectives on how to decrease treatment burden were collected through semi-structured interviews. In-depth and key informant interviews of approximately 15–25 min duration were applied to gather in-depth information using an interview guide adapted from different articles^13,20^. The feasibility of the interviews was pilot tested using face-to-face cognitive interviews with 5 patients and 5 health care providers to ensure the comprehensibility of the starting question and the usability of possible reformulations and explanations. Two nurses with master’s degree administered the qualitative interviews face-to-face in a separate quiet room which is located adjacent to the nursing head station of the cardiac clinic. The nurses involved in the interviews had ample previous experience in qualitative interview-based health researches. Besides, the main investigators provided five days training on how to effectively conduct in-depth interviews using important probing questions by strictly following the interview protocols. The nurses were trained on strict use of study criteria, explanation of study objective, obtaining written consents from participants, implementation of sampling technique, and uniform interpretation of questions. Interviews were conducted until information saturation was reached. Voice recorders and notes were used to capture information from both interviews. Finally, the results of both phase I and phase II were interpreted to provide rigorous insight into treatment burden.

### Data analysis

The quantitative data were entered into and cleaned in Epi Info version 4.6.0.2 and were exported into and analyzed in Statistical Package for the Social Sciences (SPSS) version 26. Frequencies and percentages were used for categorical variables, while mean ± standard deviation and/or median (IQR) for continuous variables. Initially, multicollinearity was checked to test correlation among the predictor variables using the variance inflation factor (VIF). A VIF < 8 was considered as a cut point for excluding collinearity. Two predictor variables called the duration of CHF and duration of CHF treatment were found collinear (VIF = 29.1) and thus one of the variables (duration of CHF treatment) was excluded from the model. The reason behind this collinearity was that almost all patients start CHF treatment immediately soon after their diagnosis.

Binary logistic regression analysis was carried out to assess the association between treatment burden and all the independent variables and to identify candidates for multivariable analysis. Independent variables with *p*-value < 0.25 in the univariable binary logistic regression analysis were re-entered into a multivariable binary logistic regression model to identify predictors of treatment burden. A *p-*value of < 0.05 was considered statistically significant.

The qualitative data was analyzed by using thematic analysis^21^. NVivo 12 qualitative data analysis software (QSR International Pty Ltd. Version 12, 2018) was used to assist the data analysis. Initially, the data was analyzed by transcribing the recorded data from Amharic into English verbatim, and then the transcript was coded and grouped into themes. Two investigators (OSM and MH) independently transcribed the audio recordings verbatim and read all participants’ notes taken during interviews to extract both patients’ and health care providers propositions to decrease burden of treatment. Coding, categorization and generation of themes were managed by the three investigators. Finally, each theme identified in phase II was transformed into a quantitative variable to analyze the relationships between the two phases. Concepts extracted from the themes were presented in narratives and triangulated with the quantitative results.

### Operational definitions

*Treatment Burden Global Score*-the sum of all items scores of the questionnaire with ‘Does not apply and missing answer considered the lowest possible score (0) [scores ranging from 0 to 150]. *No Treatment Burden*-a score of 0 for each item in the TBQ. *Low Treatment Burden*-a TBQ global score of < 59. *High Treatment Burden*-a TBQ global score of ≥ 59. *Limited Health Literacy*-if participants ‘sometimes’ or ‘often’ or ‘always’ need reading help for written health materials related to their HF. *Poor Availability of Medications*-if medications are available ‘sometimes’, ‘rarely’, ‘not at all’ as self-reported by the patient. *Poor Knowledge about HF*-if patients responded ‘insufficient’ or ‘very insufficient’ when asked about their knowledge of HF.

### Ethical approval and informed consent

The study was approved by the Ethical Review Board (ERB) of Addis Ababa University, College of Health Sciences (25/03/2021; ERB No. 259/13/2021). The study protocol was performed in accordance with the Declaration of Helsinki. The aim and protocol of the study were fully explained to all participants included in the study and written informed consent was obtained from all participants. All obtained data were treated confidentially.

## Result

### Sociodemographic characteristics of the study participants

A total of 325 patients were included in the study. The mean age of the participant was 50.47 ± 16.42 years. Out of the total studied participants, 165(50.8%) were males, 197(60.6%) were married, 87(26.8%) had completed higher education, and 216(66.5%) were from Addis Ababa. Majorities of the participant were unemployed 145(44.6%), and 242(74.5%) lived with a partner (Table 1).

**Table 1:** Sociodemographic characteristics of patients with heart failure attending cardiac clinic of Tikur Anbessa Specialized Hospital, Addis Ababa, Ethiopia, August 01–September 30, 2021 (n = 325).

Variables	Category	n (%)
Age (years) [mean ± SD = 50.47 ± 16.42]	18–30	58 (17.8)
	31–60	171 (52.6)
	> 60	96 (29.5)
Gender	Male	165 (50.8)
	Female	160 (49.2)
Marital status	Single	80 (24.6)
	Married	197 (60.6)
	Divorced	18 (5.8)
	Widowed	30 (9.2)
Education level	No formal education	54 (16.6)
	Primary school completed	98 (30.2)
	Secondary school completed	86 (26.5)
	College and above	87 (26.8)
Employment status	Unemployed	145 (44.6)
	Government employed	51 (15.7)
	Private employed	3 (0.9)
	Self employed*	88 (27.1)
	Retired	38 (11.7)
Residence	Addis Ababa	216 (66.5)
	Outside Addis Ababa	109 (33.5)
Living condition	Alone	83 (25.5)
	Cohabiting*	242 (74.5)
Presence of family support	Yes	254 (78.2)
	No	71 (21.8)
Smoking status	Never smoker	274 (84.3)
	Former smoker	47 (14.5)
	Current smoker	4 (1.2)
Monthly income (in Eth Birr) [median (IQR) = 1500 (0–5000)]	≤ 1500	170 (52.3)
	> 1500	155 (47.7)

Cohabiting*: living with a marriage partner, child, or another partner like family and/or friends, Self-employed*: farmer, daily laborer, merchant, driver, *BMI* body mass index, *Eth* Ethiopian, *IQR* inter quartile range.

### Clinical characteristics of the study participants

The majority of the participants had HF with preserved ejection fraction (HFpEF). The mean EF was 50.75 ± 15.49. The median (IQR) duration of HF diagnosis was 6 (3–10) years, and about 47.7% had NYHA class II HF. The most commonly identified underlying causes of HF were chronic rheumatic valvular heart disease (CRVHD) (42.2%), followed by ischemic heart disease (IHD) (28.3%), and then hypertensive heart disease (HHD) (13.2%) (Table 2).

**Table 2:** Clinical characteristics of patients with heart failure attending cardiac clinic of Tikur Anbessa Specialized Hospital, Addis Ababa, Ethiopia, August 01–September 30, 2021 (n = 325).

Variables	Category	n (%)
Duration of HF [median (IQR) = 6(3–10)]	≤ 5 years	159 (48.9)
	6–10 years	95 (29.2)
	> 10 years	71 (21.8)
Type of HF* (n = 306)	HFpEF	190 (58.5)
	HFmEF	52 (16)
	HFrEF	64 (19.7)
NYHA classification	Class I	13 (4)
	Class II	155 (47.7)
	Class III	106 (32.6)
	Class IV	51 (15.7)
Health literacy (frequency of needing reading help)	Never	52 (16)
	Rarely	119 (36.6)
	Sometimes	95 (29.2)
	Often	37 (11.4)
	Always	22 (6.8)
Underlying causes of HF	Chronic rheumatic VHD	137 (42.2)
	Ischemic heart disease	92 (28.3)
	Hypertensive heart disease	43 (13.2)
	Cardiomyopathies	34 (10.5)
	Myocardial infarctions	9 (2.8)
	Others*	10 (3.1)
Travel time to cardiac clinic [median (IQR) = 1.50 (1–3)]	< 1 h	57 (17.5)
	≥ 1 h	268 (82.5)
Frequency of follow up	Every month	69 (21.2)
	Every two month	60 (18.5)
	Every three month	157 (48.3)
	Every four month	19 (5.8)
	Every five month	3 (9)
	Every six month	17 (5.2)
No of appo in the last 6 month [mean ± SD = 2.70 ± 1.45]	0–2	201 (61.8)
	≥ 3	124 (38.2)
Family history of HF	Yes	50 (15.4)
	No	275 (84.6)
History of hospitalization in the past 12 months	Yes	107 (32.9)
	No	218 (67.1)
Treatment Burden	Low (acceptable burden)	287 (88.3)
	High (unacceptable burden)	38 (11.7)
Baseline EF* (n = 306)	mean ± SD = 50.75 ± 15.49	

*EF* ejection fraction, *No* number, *Appo* appointment, *NYHA* New York Heart Association, *HFpEF* heart failure with preserved ejection fraction, *HFmEF* heart failure with mid-range ejection fraction, *HFrEF* heart failure with reduced ejection fraction, *VHD* valvular heart disease, Others*: Cor pulmonale, Degenerative valvular heart disease, Congenital heart disease, Constrictive pericarditis.

### Treatment-related characteristics of the study participants

The median duration of HF treatment was 5 (2–10) years. Participants took an average of 5.01 ± 2.7 pills daily. About 70(21.5%) participants reported medication adverse effects. Most participants (51.4%) obtained their medications via the health care insurance system. Half of the participants 161(49.5%) had two or more comorbid conditions. Of which, hypertension (42.8%) was the most commonly identified comorbid condition, then followed by atrial fibrillation (27.7%), and diabetes mellitus (21.5%) (Table 3).

**Table 3:** Treatment-related characteristics of patients with heart failure attending cardiac clinic of Tikur Anbessa Specialized Hospital, Addis Ababa, Ethiopia, August 01–September 30, 2021 (n = 325).

Variables	Category	n (%)
Duration of HF treatment [median (IQR) = 5(2–10)]	≤ 5 years	167 (51.4)
	6–10 years	93 (28.6)
	> 10 years	65 (20.0)
Type of medication	Tablet/capsule only	189 (58.2)
	Injection only	5 (1.5)
	Tablet/capsule + Injection	131 (40.3)
Source of medication	Free	16 (4.9)
	Payment	142 (43.7)
	Insurance	167 (51.4)
Monthly cost of medication	Less than 500Birr	114 (35.1)
	500–1000Birr	94 (28.9)
	Above 1000Birr	117 (36.0)
Availability of medications	Always	30 (9.2)
	Often	146 (44.9)
	Sometimes	147 (45.2)
	Not at all	2 (0.6)
Presence of ADRs	Yes	70 (21.5)
	No	255 (78.5)
Knowledge about HF and its treatment	Good	274 (84.3)
	Poor	51 (15.7)
No of prescribed medications [mean ± SD = 4.73 ± 1.68]	≤ 5 Medications daily	226 (69.5)
	> 5 Medications daily	99 (30.5)
No of pills per day [mean ± SD = 5.01 ± 2.74]	≤ 5 Pills daily	205 (63.1)
	> 5 Pills daily	120 (36.9)
Presence of comorbidity	Yes	272 (83.7)
	No	53 (16.3)
Total no of comorbidities [median (IQR) = 1(1–2)]	< 2	164 (50.5)
	≥ 2	161 (49.5)
Types of comorbidities	Hypertension	139 (48.2)
	Diabetes mellitus	70 (21.5)
	Atrial fibrillation	90 (27.7)
	Dyslipidemia	34 (10.5)
	Stroke	24 (7.4)
	Thyroid diseases	8 (2.5)
	Respiratory diseases	12 (3.7)
	Cancer	10 (3.1)
	Kidney failure	19 (5.8)
	Neurologic diseases	27 (8.3)
	Gastrointestinal diseases	11 (3.4)
	Musculoskeletal diseases	17 (5.2)
	Viral infections (HBV, HIV)	9 (2.8)
	Other diseases**	57 (17.5)

*ADRs* adverse drug reactions, *No* number, *HF* heart failure, *IQR* interquartile range, *SD* standard deviation, *HIV* human immunodeficiency virus, *HBV* hepatitis B virus, Other Diseases**: Left ventricle thrombus, Schizophrenia, Erectile dysfunction, Peripheral arterial disease (PAD), Benign prostate hyperplasia, aortic aneurism.

### Description of treatment burden

Participants reported a mean global treatment burden of 27.22 (SD = 19.35) out of a possible score of 150. When analyzed individually for the five dimensions of treatment burden, the highest levels of burden were reported in administrative (mean = 7.57, SD = 7.89), financial (mean = 6.07, SD = 3.99) and lifestyle (mean = 4.94, SD = 4.98) dimensions, followed by social (mean = 4.46, SD = 5.49) and then medication (mean = 4.17, SD = 6.31) dimensions (Table 4). Overall, about 287(88.3%) of the participants reported a low burden, while only 38(11.7%) indicated a high burden.

**Table 4:** Analysis of five dimensions of TBQ among HF patients attending cardiac clinic of TASH.

Variables	Mean ± SD
**1. Medication-related burden**	**4.17 ± 6.31**
*Problems caused by the taste, shape, or size of your medication and/or discomforts caused by your injection (for example; pain, bleeding, bruising, or scars)?*	1.54 ± 2.58
*Problems caused by how many times a day you need to take your medications (for example: taking once per day/twice per day)?*	0.85 ± 2.14
*Problems caused by the efforts you need to make not to forget to take your medications (For example; not stopping taking medication when you are away from home, sorting out and using a pillbox……)?*	0.84 ± 1.86
*Problems caused by the precautions you need to take while taking your medications (for example: taking them at specific times or meals as prescribed by your doctor, not doing certain things after taking medications such as driving or lying down….)?*	0.93 ± 1.99
**2. Administrative-related burden**	**7.57 ± 7.89**
*Problems related to the time you need to spend to undergo lab tests and other related exams regularly (for example; blood tests or radiology)?*	1.89 ± 2.98
*Problems related to the time you need to spend to self-monitor your health condition regularly (for example; measuring your BP or BGL)?*	0.28 ± 1.16
*Problems related to the time you need to spend to have to go to doctor visits and other medical appointments regularly and difficulties in finding health care professionals?*	0.68 ± 1.96
*Problems related to your relationship with health care professionals during treatment (for example; feeling not listened to enough or not taken your ideas seriously)?*	0.38 ± 1.31
*Problems related to arranging medical appointments (doctor visits, laboratory tests, and other related tests) and limitation of using your time for other life events?*	1.04 ± 2.19
*Problems related to the administrative burden associated with your healthcare system provision (for example; sorting out and filling forms for hospitalization, reimbursements, and/or getting social services)?*	3.29 ± 3.70
**3. Financial-related burden**	**6.07 ± 3.99**
*Problems related to the financial burden associated with your healthcare / treatment (for example: out-of-pocket expenses or expenses not covered by insurance)?*	6.07 ± 3.99
**4. Lifestyle change-related burden**	**4.94 ± 4.98**
*Problems related to making dietary changes as recommended by your doctor (for example; avoiding certain foods like salty foods, reducing alcohol intake, stopping smoking…)?*	3.12 ± 3.31
*Problems related to following doctors physical exercise recommendation (for example: walking, jogging, swimming…)*	1.83 ± 2.69
**5. Social life-related burden**	**4.46 ± 5.49**
*Problems related to the impact of your treatment on your social life (for example: seeking help from family, friends and other people in your daily life, being embarrassed to take your medication in public…)?*	1.92 ± 3.06
*“The need for regular medical healthcare reminds my health problems.”*	2.54 ± 3.46

### Factors associated with treatment burden

Using binary logistic regression analysis, patients with HF with low treatment burden and high were compared using the sociodemographic, disease and treatment-related characteristics. Accordingly, presence of two and more comorbidities (Crude odds ratio [COR] = 2.81, 95% confidence interval [CI]: 1.34–5.87), more than five prescribed medications (COR = 2.02, 95%CI: 1.02–4.03), daily intake of more than five pills (COR = 3.41, 95%CI: 1.69–6.89), presence of ADRs (COR = 3.57, 95%CI: 1.77–7.24), and poor availability of medications (COR = 3.32, 95%CI: 1.59–6.95) were significantly associated with higher treatment burden. Conversely, family support (COR = 0.43, 95%CI: 0.21–0.87) was negatively associated with a higher treatment burden (Table 5).

**Table 5:** Univariable and multivariable logistic regression analysis of factors associated with treatment burden among patients with heart failure attending adult cardiac clinic of Tikur Anbessa Specialized Hospital, Addis Ababa, Ethiopia, August 01-September 30, 2021 (n = 325).

Variables	Treatment Burden		COR (95% CI)	*p* value	AOR (95% CI)	*p* value
	Low, n (%)	High, n (%)				
**Education**						
No formal education	47 (87.0)	7 (13.0)	1	1	1	1
Primary school com	92 (93.9)	6 (6.1)	0.44 (0.14–1.38)	0.158	2.05 (0.45–9.34)	0.352
Secondary school com	78 (90.3)	8 (9.3)	0.69 (0.24–2.02)	0.497	3.18 (0.59–17.08)	0.178
College and above	70 (80.5)	17 (19.5)	1.63 (0.63–4.24)	0.315	6.66 (1.16–38.43)	0.034*
**Occupation**						
Retired	33 (86.8)	5 (13.2)	1	1	1	1
Govern’t Employed	41 (80.4)	10 (19.6)	1.61 (0.51–5.17)	0.424	1.91 (0.44–8.38)	0.390
Private Employed	2 (66.7)	1 (33.3)	3.30 (0.25–43.47)	0.364	2.42 (0.12–47.75)	0.563
Self employed	85 (96.6)	3 (3.4)	0.23 (0.05–1.03)	0.055	0.19 (0.03–1.11)	0.065
Unemployed	126 (86.9)	19 (3.1)	0.99 (0.35–2.86)	0.993	1.81 (0.40–8.21)	0.439
**Knowledge of HF**						
Good	42 (82.4)	9 (17.6)	1	1	1	1
Poor	245 (89.4)	29 (10.9)	0.55 (0.24–1.25)	0.154	0.49 (0.13–1.90)	0.303
**Family support**						
No	57 (80.3)	14 (19.7)	1	1	1	1
Yes	230 (90.6)	24 (9.4)	0.43 (0.21–0.87)	0.020	0.42 (0.17–1.04)	0.061
**No of comorbidities**						
< 2	153 (93.3)	11 (6.7)	1	1	1	1
≥ 2	134 (83.2)	27 (16.8)	2.81 (1.34–5.87)	0.006	2.74 (1.02–7.39)	0.047*
**Diabetes mellitus**						
No	229 (89.8)	26 (10.2)	1	1	1	1
Yes	58 (82.9)	12 (17.1)	1.82 (0.87–3.83)	0.113	0.72 (0.24–2.13)	0.548
**Travel time to clinic**						
< 1 h	53 (93.0)	14 (7.0)	1	1	1	1
≥ 1 h	234 (87.3)	14 (12.7)	1.93 (0.66–5.66)	0.234	1.66 (0.46–6.06)	0.443
**No of appo in 6mon**						
0–2	183 (91.0)	18 (9.0)	1	1	1	1
≥ 3	104 (83.9)	20 (16.1)	1.96 (0.99–3.86)	0.054	1.53 (0.64–3.63)	0.340
**Total prescribed med**						
≤ 5 Medications	205 (90.7)	21 (9.3)	1	1	1	1
> 5 Medications	82 (82.8)	17 (17.2)	2.02 (1.01–4.03)	0.045	0.47 (0.13–1.63)	0.231
**Total no of pills/day**						
≤ 5 Pills	191 (93.2)	14 (6.8)	1	1	1	1
> 5 Pills	96 (80.0)	24 (20.0)	3.41 (1.69–6.89)	0.001	7.38 (2.23–24.41)	0.001*
**Medication cost**						
< 500Birr/month	109 (95.6)	5 (4.4)	1	1	1	1
500–1000Birr/month	80 (85.1)	14 (14.9)	3.82 (1.32–11.03)	0.013	6.09 (1.61–23.05)	0.008*
> 1000Birr/month	98 (83.8)	19 (16.2)	4.23 (1.52–11.75)	0.006	5.29 (1.46–19.18)	0.011*
**Drug availability**						
Good	165 (93.8)	11 (6.2)	1	1	1	1
Poor	122 (81.9)	27 (18.1)	3.32 (1.59–6.95)	0.001	3.33 (1.33–8.36)	0.010*
**Presence of ADR**						
No	234 (91.8)	21 (8.2)	1	1	1	**1**
Yes	53 (75.7)	17 (24.3)	3.57 (1.77–7.24)	< 0.001	4.04 (1.63–10.03)	0.003*

*COR* crude odds ratio, *AOR* adjusted odds ratio, *CI* confidence interval, *Govern’t* government, *Appo* appointment, *HF* heart failure, *ADR* adverse drug reaction, *No* number, *mon* month, *Med* medication, *com* completed.

*significant at *p* < 0.05.

Significant values are in bold.

On further multivariable binary logistic regression model; a higher education level (adjusted odd ratio [AOR] = 6.66, 95%CI: 1.16–38.43), presence of two and more comorbidities (AOR = 2.74, 95%CI: 1.02–7.39), daily intake of more than five pills (AOR = 7.38, 95%CI: 2.23–24.41), presence of ADRs (AOR = 4.04, 95%CI: 1.63–10.03), poor availability of medications (AOR = 3.33, 95%CI: 1.33–8.36), and higher monthly costs of medication (500-1000Birr [AOR = 6.09, 95%CI: 1.61–23.05] and more than 1000Birr [AOR = 5.29, 95%CI: 1.46–19.18]) were found to be predictors of higher treatment burden (Table 5).

### Qualitative analysis of patients’ and health care providers propositions to decrease treatment burden

Patients' and health care providers propositions on how burden of treatment could be mitigated were categorized into three major themes: (1) propositions related to improving self-care management, (2) propositions related to improving the structural organization of the clinic and the hospital, and (3) propositions related to improving the healthcare system provision. Then, subthemes emerged in each theme (see Supplementary Tables S1 and S2).

### Theme 1: improving self-care management

Most participants requested for modification to pharmacological treatment to improve their self-care management. For instance, changing regimen with smaller pills, pills with a better taste, medications requiring a less strict dosage schedule, etc.

Regarding the challenge associated with the treatment regimen, one of the participants who had history of taking eleven pills per day underscored that:

I am tired of ingesting so many pills daily. I have taken too many medications for the last eight years. At times, I become confused about the number of tablets I am taking. In such difficult times, I wished that my doctor simplified my treatment regimen (P-1).

Interestingly, majority of the physicians suggested that increasing the procurement of medications with a fixed dose combination (FDC) is helpful to reduce the medication burden of patients.

Availing FDC medications in various governmental hospitals and Kenema pharmacies at a reasonable price not only prevents medication related burden but also financial related burden that occurs secondary to pill burden (HCP-3, chief resident).

Patents having higher education proposed that changing consultation content is vital for enhancing their self-care efficacy. In this regard, one participant with a BSc degree asserted that:

I personally expect physicians to provide more information about the care I am receiving, the likely evolution, sign and symptoms of my medical condition along with the treatment recommendations, and/or adverse effect profiles associated with my treatments (P-7).

To this end, an experienced cardiac fellow specialist serving in the cardiac clinic supported the patients concern as follow:

I think counseling on adherence and informed choice of patients and their involvement in treatment decisions is easy to implement than just prescribing and telling the patient to take medication (HCP-1, cardiac fellow specialist).

Unique to patients’ propositions, some health care providers proposed the establishment of patient support group systems as one possible method of boosting self-care management.

I believe that creating support group systems to meet and discuss with other older and experienced HF patients to share their tips and methods to live with HF and its care would be helpful to strengthen interaction between patients (HCP-5, senior resident).

### Theme 2: improving structural organization of the clinic and hospital

Major issues described by most participants as a means of structural improvement were related to waiting area and period, patient load, and availability of medications and laboratory tests.

With regards to improving waiting area and period, one participant underscored that:

The waiting area of the clinic is too congested and the waiting period before seeing the physician is too long. I wish there were enough seats in the waiting room where I can find my physicians easily on time (P-2).

Patients and health care providers believe that one of the barriers for poor structural organization is the tightness of follow-up schedule. Patients recommend that extending the follow-up schedule is important to prolong the consultation time needed to undergo complete check-up.

To my personal judgment, you get explicit ambulatory care service only in the beginning of the follow-up session. In the final times, most doctors often get bored and exhausted to provide denotative health care service. To minimize patients upset and accommodate high patient load, it is good to change the follow up schedule from half day to full day (P-6).

Majority of the participants considered the frequent changing of physicians during each follow up session as a substantial contributing factor for their administrative related burden.

There is change of physicians during each follow-up appointment. If so, it would be difficult to consistently assess my physical change and clinical progress as everything may not be recorded in the electronic system. Besides, I would be psychologically satisfied when I get follow-up service with a permanent doctor (P-1).

With respect to improving structural organization of the clinic and the pharmacy, one of the nurses who had 11-year work experience in the clinic asserted that:

If possible, the clinic and the examination room should be located at a close proximity so as to avoid the discomfort caused by the process of traveling. The same principle should be employed for the case of the hospitals OPD pharmacy and the health insurance office (HCP-6, nurse with eleven-year work experience).

Health care providers emphasized that establishing a separate pharmacy in the vicinity of the clinic is another proficient method to lessen the administrative related burden imposed on patients.

If possible, because the cardiac clinic is one of the heavy burdened clinics in TASH, it is good to establish its own separate pharmacy for the clinic where only cardiac medications will be kept and dispensed (HCP-1; cardiac fellow specialist).

### Theme 3: improving health care system provision

Most participants suggested on the need of controlling harsh relationship between patients and non-medical staffs to strengthen the quality of the health care provision.

I have good relationship with the doctors but my problem is with pharmacists, nurses’ and guards’ behavior. Always human being needs to be monitored. This is because ‘a hoarse without a poll and a man without a discipline are similar’ (P-14).

As per the patient’s perspective and witnessed by their physicians, the health insurance system has a lot of problems and requires thorough revision.

I get health insurance because I have a financial problem to buy medications. Regrettably, I couldn’t even access medications via the health insurance system. I believe that the health insurance system needs to be organized, controlled, and pragmatically implemented (P-5).

Obtaining financial help is another issue raised by patients to comply with their financial reality.

As a result of financial constraint, I personally prefer eating fruits and vegetables over undergoing expensive laboratory test like INR. I would like a financial help to pay for the tests. Otherwise, I can’t afford the INR test which is frequently asked by the doctors (P-2).

From the health care providers perspective, manufacturing medications locally and preparing sponsorships improves the health care provision system.

Manufacturing medications at local settings and preparing sponsorships for financially limited patients will be helpful to alleviate treatment burden (HCP-10, R3 resident).

## Discussion

This study is the first to profoundly assess treatment burden among patients with HF in Ethiopia from both patients and health care providers’ perspectives using a mixed methods study approach. Because the pursuit of conceptualizing and measuring treatment burden is relatively novel, there are currently only a few studies to compare our results to.

The present study finding reported a mean global treatment burden of 27.22 (SD = 19.35). This finding is congruent with the finding of a study conducted in Switzerland (M = 26.8, SD = 18.6)^22^. However, it is lower than the studies done in USA (M = 37.01, SD = 24.45)^23^, Australia (M = 56.5, SD = 34.5)^8^, Qatar (Median = 40.5, IQR = 38)^24^, Côte d’Ivoire (M = 33.3, SD = 19.6)^13^, and higher than a study done in USA [Clevland] (M = 22.84, SD = 24.57)^25^. The discrepancy could be attributed to the differences in the nature of the health care provided, fragmented healthcare system provision, and economic factors. Moreover, the variation could also be because the current study measured the level of treatment burden only from the perspective of CHF population alone.

According to the finding of this study, the highest levels of treatment burden were reported in the administrative (mean = 7.57, SD = 7.89), financial (mean = 6.07, SD = 3.99) and lifestyle (mean = 4.94, SD = 4.98) dimensions, followed by social (mean = 4.46, SD = 5.49) and medication (mean = 4.17, SD = 6.31). This finding is incongruent with studies conducted in Australia^8^ that reported the highest treatment burden on financial, lifestyle, social, administrative, and medication dimensions, respectively, and in Qatar^24^ that showed the highest treatment burden on medication, lifestyle, administrative, social and financial dimensions, respectively. The incongruity could be attributed to the differences in the nature of the health care provided, fragmented healthcare system provision, and economic-related factors. For instance, Qatar provides free ambulatory healthcare services.

The present study finding revealed that about 287(88.3%) of participants reported low burden, and only 38(11.7%) indicated high burden. This finding is incongruent with Tran’s et al.^26^ study, where approximately 47% experienced low burden, 28% moderate burden, and 24% high burden. The possible reason for this discrepancy could be due to differences in the characteristics of study subjects. For instance, Tran's sample was larger (n = 502), hospitalized, slightly older (mean = 59.3; SD = 17.0), and highly educated (35%). The other reason for this variation is the difference in the statistical method employed and the cut-off score used to categorize patients as low and high burden. Previous studies approached TBQ as a continuous variable unlike ours which considered it as a dichotomous categorical variable. Nevertheless, this finding is in agreement with a study by Pedersen et al.^27^ which revealed that 13% of patients exhibited a higher treatment burden.

One of the major findings of this study was that patients who had higher education achievement tend to have a higher burden of treatment compared with those who had no formal education. This finding is consistent with a study by Herzig et al*.*^22^. However, it is incongruent with a study by Al-mansouri et al.^24^ that revealed less educated patients have a higher burden of treatment and lower quality of life. This incongruity might be ascribed to the concept that being highly educated doesn’t necessarily depict the level of health literacy and knowledge about an illness.

The result of this study indicated that patients who had two and more comorbid conditions have a higher treatment burden compared with their counterparts. This finding is in keeping with previous studies by Sav et al*.*^8^, Morris et al*.*^28^, Al-mansouri et al*.*^24^, and Schreiner et al*.*^25^, which showed that having extra chronic conditions leads to a higher perceived treatment burden.

The finding of this study demonstrated that a more daily intake of pills (≥ 5) was strongly associated with a higher treatment burden. A similar observation was noted in a study by Morris et al*.*^28^. This study has unveiled that poor availability of medications, presence of ADR, and high monthly cost of medications were predictors of a higher treatment burden. In fact, these findings are new.

The qualitative finding of this study revealed that patients proposed improvement in their self-care management, structural organization of the clinic and/or the hospital, and health care system provision to minimize treatment burden. This finding is consistent with previous studies by Ting et al.^29^ and Tran et al*.*^13^. Similarly, this study found that health care providers' propositions on decreasing treatment burden conform with patients’ perspectives but with more considerable emphasis on medical-related factors. A similar observation was noted in previous studies^13,22^.

Finally, this study has some limitations. First, it was cross-sectional and thus captured treatment burden at a single point in time. Second, some variables such as smoking habit, duration of HF, and number of pills were gathered directly from the patients, which may be subjected to both social desirability and recall bias. Third, quantitative data collection was done through an interviewer administration approach that may lead to social desirability bias. To ameliorate this, data collectors strictly followed the interview protocols. Fourth, the fact that sample being relatively younger to the general age of individuals with heart failure could have affected the generalizability of findings. Fifth, this study was trammeled to patients with HF who could speak Amharic language only and thus may not be generalized to other cultures or countries.

## Conclusion

The findings of this study demonstrated that a substantial proportion of patients faced low levels of treatment burden. Our findings revealed that higher education, presence of two and more comorbidities, more than five pills daily intake, poor medications availability, presence of ADRs, and monthly expenses of medication were found to have a statistically significant association with high treatment burden. The qualitative findings of this study unveiled that improving self-care management, structural organization of the clinic, and the healthcare provision had paramount importance to minify treatment burden. Therefore, factors that increase treatment burden should be considered when designing healthcare interventions for patients with HF.

## Supplementary Information

Supplementary Information 1.

Supplementary Information 2.

## Data Availability

The datasets used and/or analyzed during the current study are available from the corresponding author upon reasonable request.

## Abbreviations

ADRs: Adverse drug reactions
AOR: Adjusted odds ratio
CHF: Chronic heart failure
COR: Crude odds ratio
ERB: Ethical Review Board
FDC: Fixed-dose combination
HF: Heart failure
ISPOR: International Society for Pharmacoeconomics and Outcomes Research
MRT: Mapi Research Trust
TASH: Tikur Anbessa Specialized Hospital
TBQ: Treatment Burden Questionnaire
VIF: Variance inflation factor
