# Supplementary material for: Treatment burden among patients with heart failure attending cardiac clinic of Tikur Anbessa Specialized Hospital: an explanatory sequential mixed methods study
Source: Sci Rep. 2022 Nov 7;12:18899. doi: 10.1038/s41598-022-23700-0 (PMC9640606; doi:10.1038/s41598-022-23700-0)
Supplement: Supplementary file 2 — Supplementary Information 2. [file 41598_2022_23700_MOESM2_ESM.docx]

**Treatment Burden among Patients with Heart Failure Attending Cardiac Clinic of Tikur Anbessa Specialized Hospital, An Explanatory Sequential Mixed Methods Study**

Minimize Hassen^1^ , Desalew Mekonnen^2^ , Oumer Sada Muhammed ^2*^

**Supplementary Table S1. Patients’ Propositions to Decrease their Burden of Treatment**

| Theme-1: Propositions related to improving self-care management | |
| --- | --- |
| Subtheme 1: Modifications in the pharmacological treatment | |
| P-1 | *I am tired of ingesting so many pills daily. I have taken too many medications for the last eight years. At times, I become confused about the number of tablets I am taking. In such difficult times, I wished that my doctor simplified my treatment regimen.* |
| P-2 | *Because some of the pills I took have an unpleasant taste and are causing me severe epigastric pain, my motivation to take my medication has decreased over time. Hence, I prefer to take pills with pleasant tastes and have minimal side effects.* |
| Subtheme 2: Changes in the consultation content | |
| P-7 | *I expect physicians to provide more information about the care I am receiving, the likely evolution, sign and symptoms of my medical condition along with the treatment recommendations, and/or adverse effect profiles associated with my treatments.* |
| P-11 | *Whenever my medications are modified, physicians don’t tell me the exact reason why they were changed in the course of therapy. At least, I deserve an explanation.* |
| P-12 | *I don’t want to be ashamed whenever I come to the cardiac clinic for follow-up service. As a result, I want more awareness campaigns so that I would no longer be estranged.* |
| **Theme-2: Propositions related to improving the structural organization of the clinic and the hospital (TASH)** | |
| **Subtheme 1: Improving waiting area and period** | |
| P-3 | *The hospital OPD Pharmacy is the slowest service. I’d wish that there were enough seats in the waiting room which can accommodate the high flow of patients.* |
| P-2 | *The waiting area of the clinic is too congested and the waiting period before seeing the physician is too long. I wish there were enough seats in the waiting room where I can find my physicians easily on time.* |
| **Subtheme 2: Improving the availability of medication in Hospital** | |
| P-1 | *Surprisingly, except for the cheapest medications, almost all the other medications are not available at the OPD pharmacy. I am being supportive of ordinary people’s critics that pharmacists working in hospitals sell medications to private pharmacies to boost their economy. Prove me wrong or otherwise, concerned regular authorities should control such misconduct and take action accordingly.* |
| P-9 | *Even if medications are available at the pharmacy, the approach of the dispensing pharmacists is often annoying. Besides, when all prescribed medications aren’t available in a single pharmacy, collecting from different pharmacies is tiresome due to transportation problems. To overcome this, medications must be adequately available in pharmacy stores.* |
| P-5 | *In recent days, pharmacists working in Kenema pharmacy return patients by stating that they are instructed by higher officials not to dispense brand medications to patients coming with health insurance. Because of this, I am becoming less adherent to my medications and my health condition is subsequently deteriorating.* |
| **Subtheme 3: Minimizing Patient load** | |
| P-6 | *To my judgment, you get explicit ambulatory care service only at the beginning of the follow-up session. In the final times, most doctors often get bored and exhausted to provide denotative health care services. To minimize patients' upset and accommodate the high patient load, it is good to change the follow-up schedule from half day to a full day.* |
| P-9 | *Because HF patients are increasing from time to time, it is better to increase the number of physicians in each follow-up and extra rooms must be built.* |
| P-10 | *For physicians to effectively listen to patients’ complaints and provide ambulatory service, the crowd must be reduced. Otherwise, it will be a copy-paste health care delivery.* |
| **Subtheme-4: Improving frequent changing of physicians during each follow-up** | |
| P-1 | *There is a change of physicians during each follow-up appointment. If so, it would be difficult to consistently assess my physical change and clinical progress as everything may not be recorded in the electronic system. Besides, I would be psychologically satisfied when I get follow-up service with a permanent doctor.* |
| P-6 | *I have a good relationship with physicians but the problem is I do not get the doctor I found today in my next follow-up appointment.* |
| **Subtheme-5: Availing fully functional laboratory tests in the hospital** | |
| P-12 | *What I heard from the national media is the donation of medical devices and laboratory equipment from international institutions to various referral hospitals including TASH, but what I am witnessing on the ground is the opposite. For instance, it had been almost 2 years since this hospital stopped doing the International Normalized Ratio (INR) test. As much as possible, important laboratory tests like INR should be available and must be made functional in the hospital.* |
| P-2 | *The queue for undergoing echocardiography along with the shortage of the device poses a great challenge for performing the test regularly in each appointment.* |
| **Theme-3: Propositions related to improving health care system provision** | |
| **Subtheme-1: Social support** | |
| P-3 | *Since I am sick and I can’t find a job by myself, the government should help me either by providing financial aid or by creating job opportunities that would help me maintain my family and professional life.* |
| P-13 | *Preparing temporary shelters in the hospital could be helpful for patients who travel a long distance to reach the clinic, and for patients who come from outside Addis Ababa but have no relatives in the city. For instance, it would enable them to save money and protect them from unexpected thefts.* |
| **Subtheme-2: Improving the relationship between non-medical staff members and patients** | |
| P-6 | *The attitude of the guards assigned in the waiting room should be strictly followed by the respective concerned body because they make the environment uncomfortable due to their poor communication skills with patients. Surprisingly, some of them even try to dishonor us and act more than the health care workers.* |
| P-9 | *The non-medical staff members bring patients of their own to the follow-up without waiting for a queue. I also witnessed the same act with the medical staff members. These kinds of actions should be corrected immediately as they are becoming serious causes of conflict.* |
| P-14 | *I have a good relationship with the doctors but my problem is with pharmacists, nurses, and guards’ behavior. Always human being needs to be monitored. This is because a horse without a poll and a man without a discipline are similar.* |
| **Subtheme-3: Strengthening and organizing the health insurance system** | |
| P-3 | *I deliberately modified my self-care autonomously in absence of my physician's input to just comply with my financial reality.* |
| P-5 | *I get health insurance because I have a financial problem to buy medications. Regrettably, I couldn’t even access medications via the health insurance system. I believe that the health insurance system needs to be organized, controlled, and pragmatically implemented.* |
| P-2 | *As a result of financial constraints, I prefer eating fruits and vegetables over undergoing expensive laboratory tests like INR. I would like financial help to pay for the tests. Otherwise, I can’t afford the INR test which is frequently asked by the doctors* |
| P-6 | *To tell you frankly, I took my medication only when I have money to buy it. My monthly salary is not adequate even to buy oil and teff, let alone the medications* |

**Supplementary Table S2. Health Care Providers' Propositions on How to Decrease Patients Perceived Burden of Treatment**

| **Theme-1: Propositions related to improving self-care and modifying HF regimens** | |
| --- | --- |
| **Subtheme-1: Improving counseling tips during consultations** | |
| HCP-1 | *Physicians should provide a detailed explanation for patients about appropriate medication administration instead of prescribing similar medications all the time.* |
| HCP-2 | *I think counseling on adherence and informed choice of patients and their involvement in treatment decisions is easy to implement than just prescribing and telling the patient to take medication regardless of trying to address factors that might affect adherence. Prescribed and dispensed doesn’t necessarily mean the medication is taken”* |
| HCP-3 | *The prescribing physician and dispensing pharmacist should counsel patients regarding drug-food interactions along with necessary precautions to be followed.* |
| **Subtheme-2: Providing Health Education** | |
| HCP-4 | *Physicians and patients should take adequate time to discuss the patient's health problem in concert with their treatment modalities until it is fully understood. Furthermore, health education should be provided to HF patients at the ambulatory clinic.* |
| **Subtheme-3: Creating patient support group systems** | |
| HCP-5 | *I believe that creating formalized groups or support group systems to meet and discuss with other older and experienced HF patients to share their tips and methods to live with HF and its care would be helpful to strengthen the interaction between patients.* |
| **Subtheme-4: Availing fixed-dose combination (FDC) medications** | |
| HCP-6 | *Fixed dose-based medicine preparations should be procured to decrease pill burden, which is one basic component of the medication-related burden* |
| HCP-3 | *Availing fixed-dose combination (FDC) medications in various government hospitals and Kenema pharmacies at a reasonable price not only prevents medication-related burden but also a financial related burden that occurs secondary to pill burden.* |
| **Theme-2: Propositions related to improving the structural organization of the clinic and the hospital (TASH)** | |
| HCP-6 | *If possible, the cardiac clinic and the examination room should be located at a similar site or in near proximity to avoid the discomfort caused by the process of traveling. The same principle should be employed in the case of the hospital's OPD pharmacy and the health insurance office.* |
| HCP-7 | *Strengthening the supply of medications, making the follow-up schedule on a full-day basis, availing laboratory tests in the hospital, and making them fully functional are indispensable to alleviate patients’ treatment burden.* |
| HCP-8 | *Some patients believed that the consultation time isn’t sufficient to undergo a complete checkup and as a result, they sometimes go to their home with minimal satisfaction with the ambulatory care service they received. The only way to surmount such frequent complaints is by extending the follow-up schedule.* |
| HCP-1 | *If possible, because the cardiac clinic is one of the heavily burdened clinics in TASH just like the endocrine and diabetic clinic, it is also good to establish a separate pharmacy for the clinic where only cardiac medications will be kept and dispensed.* |
| HCP-1 | *To the minimum, cheaper essential medications must be routinely available in hospitals. Patients with health insurance should be warmly welcomed in Kenema pharmacies and should not be denied from accessing their medications.* |
| **Theme-3: Propositions related to improving health care system provision** | |
| **Subtheme-1: Controlling the link process to the cardiac clinic** | |
| HCP-6 | *The linking process of patients to the cardiac clinic should be done properly but not via social or direct transfer from the emergency department. Once resolved, some admitted patients may need to be referred out to be seen in primary care hospitals.* |
| **Subtheme-2: Establishing a well-organized health insurance system** | |
| HCP-9 | *Establishing a well-organized health insurance system, sufficiently availing medications in Kenema pharmacies at every corner of the country, improving patients’ economy, and widely establishing pharmacies that provide service at free or discount would have paramount importance for minimizing patients’ treatment regimen fatigue.* |
| **Subtheme-3: Social support** | |
| HCP-5 | *Creating opportunities for patients to obtain medications through sponsorship or health insurance system would be helpful to alleviate the financial burden of patients.* |
| HCP-10 | *Manufacturing medications in local settings and preparing sponsorships for financially limited patients will be helpful to alleviate treatment regimen fatigue.* |
